# Communication Mechanisms and Implications of the COVID-19 Risk Event in Chinese Online Communities

**DOI:** 10.3389/fpubh.2022.809144

**Published:** 2022-07-19

**Authors:** Li Pengpeng, Zhong Fangqi, Zhuo Qianru

**Affiliations:** ^1^Shiliangcai Journalism and Communication School, Zhejiang Sci-Tech University, Hangzhou, China; ^2^School of Communication, Fujian Normal University, Fuzhou, China; ^3^College of Communication, National Chengchi University, Taipei, Taiwan

**Keywords:** uncertainty, risk communication, risk perception, COVID-19, propagation mechanisms

## Abstract

Based on the context of communication and use of online communities in China, this study explored the characteristics and defects of risk communication of the government and official media in the event of COVID-19, as well as the factors that affected people's perception of the risk and protective behavior. The following results were found: (1) The government and official (mainstream) media accounts suffered from information lag in the early stage of COVID-19, while self-media accounts played the role of risk sensors, which caused people to have less trust in the government and the authorities and turn to the truth on self-media accounts. However, the low accessibility of self-media accounts and the imperfect check mechanism provided a hotbed for rumors, which further led to more fear and worry about risks. (2) During the middle and later periods of COVID-19, the government and the official media began to pay attention to the influence of self-media on peoples' emotions and behavior, and gradually improved the supervision of online information and the operation of official media accounts. This is intended to achieve information consistently and link mechanisms between official media and self-media to prevent and correct mistakes, as well as to achieve effective risk communication of information transparency, opinion exchange, and public sentiment stabilization.

## Background

On 30 January 2020, the WHO declared COVID-19 a public health emergency of international concern (1), and the global community continues to struggle with the risk today as the virus continues to mutate. This risk event has had far-reaching health and economic impacts across the globe, resulting not only in a severe death toll but also a range of social and psychological responses (2).

It is indisputable that the public's risk perception cannot be separated from the media's construction and discourse of risk issues. The most important media include traditional media, social media, and interpersonal communication channels. However, the media's reporting and communication frameworks are inevitably affected by the values, organizational constraints, or degrees of expertise of the communicators, which may lead to a lack of objectivity and accuracy, thus affecting the risk perceptions of the people receiving the information. When sudden risks and crises occur, timely news coverage is crucial. People rely on the media to obtain timely, up-to-date, and important risk information to prevent exposure to risks (3). A study by Frewer et al. (4) points out that professional news reporting may also trigger associated secondary risks during risk communication, but governments responsible for risk management and communication often fail to recognize or properly address the problem. This can lead to increased reporting of risk events and the resulting public outrage without providing any support for risk resolution.

The Internet and social media have quickly become a major source of public information for risk and behavioral response. Any organization or individual can take advantage of the rapid dissemination and imperfect censorship of the Internet to publish their observations and opinions with the intention of gaining more recognition, attention, and even benefits. As Beck (5) argues regarding the nature of communication in modern risk societies, no one can claim to be an expert on the characteristics and hazards of risk, but everyone can assume expertise by constructing and interpreting risk based on their own experience and understanding.

Based on the communication characteristics of different media, governments have the responsibility to release risk warnings, assessments, and prevention information to the public in a timely manner when a risk event occurs, to prevent or correct wrong information to ensure the effective and accurate dissemination of risk information and social stability, and to avoid social panic and overreactions by the public. Huang (6) found that when the government takes a proactive response and conducts effective risk communication with the public when a crisis event occurs, it can effectively reduce the occurrence of unnecessary risks. The same conclusion was obtained by Su and Chen (7). Fetzer et al. (8) confirms that a government's response to risks and coping strategies can affect people's risk perception and feelings. If a government's response to risks is insufficient, it will cause a more negative emotional response from the people, which hinders risk management and response and even make the scope of influence of the risk event wider and more harmful.

Considering that the public is the direct victim of risk events, their risk perceptions often show more complex dynamics in the diverse discussions of experts, governments, and media, and the perceived risk attributes and risk hazards will affect its behavioral responsiveness and risk management measures.

From this background, this research proposes the following research questions:

**Research question 1:** What was the risk communication role of official media accounts vs. self-media accounts in the early stages of the COVID-19, and what were the characteristics and differences?
**Research question 2:** What were the omissions and remedial measures in risk communication and management by the official media during the risk event?
**Research question 3:** What are the characteristics of public perceptions of risk emergencies and what factors influence them?

In response to these questions, this study argues that reflections on the early communication mechanism and mid- and late-stage management practices of risk events can provide important reference points for effective risk management and public communication in the future.

## Risk Communication and Perception

Risk is a complex concept with multiple attributes, with uncertainty as its main characteristic (Beck, 1992). Uncertainty represents a sense of possibility or likelihood (9), which refers to the probability of occurrence, the time of occurrence, the consequences of risk, the scope and magnitude of impact (10), and the factors that cause it (11, 12) and the uncertainty of people's ability to cope with the risk (11, 13). These can include natural, social, political, economic, and technological risks.

Beck (1992) sees risk as a part of social culture, which represents not only a cognitive system but also a high degree of uncertainty and artificial constructs, representing a potential threat and disaster. Babrow et al. stated in their problematic integration theory that people will experience uncertainty, and the related awareness and behavior will be biased when the details of a problem situation are vague, complex, unpredictable, or presented as probabilistic events when there is a lack of necessary cognitive information or inconsistent information content, and when people feel unstable and unbalanced about their knowledge state or the overall knowledge state (14). Therefore, the communication and management of risk must be based on the premise that these uncertainties can be effectively assessed and managed (15).

Moreover, common people's perception of risk and their behavioral decision paths are often different from those of scientists, who are not only guided by the framework of media reports, or influenced by more subjective aspects, such as values, psychology, emotions, and interpersonal communication. At the media level, the media-system dependency theory states that in times of risk crises with high uncertainty, the public increases its reliance on the media and tends to use media they perceive as trustworthy for risk assessment and risk response advice (16) and that the degree of trust people place in different media can significantly influence people's emotions and risk perceptions.

Although previous studies have generally confirmed the influence of traditional media (17) and interpersonal communication (18), including television, newspapers, and magazines, on people's risk perception, the popularity of the Internet and the rapid development of social media have become an important channel for people to obtain risk information (19). Previous studies showed that during the MERS outbreak in Korea, social media became the main channel through which people obtained information about the risk. The more often they were exposed to social media, the more they perceived the risk as having high threat and susceptibility (20).

Similarly, in the COVID-19 outbreak, the Lancet (2020) reported that social media was one of the main sources of timely COVID-19 risk information for the population. However, it has also been shown that during the epidemic, the public's trust in traditional media, especially television, rebounded and they were particularly inclined to obtain risk information from traditional media (21).

Ning et al. (2) stated that, in unexpected risk events like COVID-19, the consistency of risk information disseminated by different media channels is crucial for people to correctly perceive and respond to risks. However, during the COVID-19 pandemic, due to the novelty of the risk and the slow pace of scientific research on its causes and prevention, there has been an influx of (sometimes contradictory) risk information and misleading news in traditional and social media (22, 23). Misinformation on how to prevent and combat misinformation about this risk is also proliferating, posing a serious obstacle to individual health management and social risk governance (24). The analyses and studies of the health status of the population during this risk event in different countries have confirmed that, due to the lack of knowledge about the new risks (25), the reduced social connectedness (26–28), and other factors, people generally show negative emotions such as fear, anxiety, and worry (29).

In terms of the correlation between emotion, risk perception, and behavior, the risk as feeling hypothesis suggests that people follow both cognitive (rational system) and emotional (empirical system) paths when making risk assessments and that emotions generally exert more influence on subsequent attitude formation and behavioral decisions (30). Risk communication has long used fear appeals to arouse people's attention to specific risks in order to guide them to adopt the right behaviors to reduce risk harm.

Risk communication scholars have further examined the dialectical relationship between fear emotions and people's risk perceptions and behaviors and found that, on the one hand, fear can raise the importance of risk issues (29) and help motivate risk-responsive behaviors (31), while on the other hand, if fear is too strong or persistent for a longer period of time, people tend to lose control over managing risk or tend to question the effectiveness of preventive behaviors (32, 33).

Ramkissoon and Smith (34) and Van Bavel et al. (35) suggest that fear appeals are effective in motivating risk coping behaviors only when people are given a strong sense of efficacy (e.g., their ability to reduce or combat risk). Ramkissoon (26–28) also demonstrated that discussions among friends and family about issues related to the risk of a COVID-19 can alleviate people's negative emotions caused by risk uncertainty. In addition, a stable or positive emotional state is more likely to facilitate individual and collective action to ultimately reduce the risk of harm. Therefore, instead of discussing whether negative emotions can stimulate people's risk perceptions and behaviors, we should instead explore the information dissemination mechanisms that influence people's emotions or risk perceptions, and how to stabilize people's emotions to achieve effective risk communication and management.

Given the complexity of common people's risk perception and behavior, some scholars suggest that all stakeholders must, based on a full understanding of the characteristics of risk and the characteristics of the public's risk perception, communicate and interact promptly and effectively with each other regarding the existence, nature, formation, severity, affordability, and other relevant messages of risk. The aim is to inform people about the characteristics of risks and any preventive measures and increase their awareness, thereby reducing the negative psychology of fear, powerlessness, or numbness (36–38). Stabilizing people's emotions to guide their individual or collective actions to mitigate risks requires mediating conflicts between stakeholders and developing more specific and effective risk management strategies (37). In other words, effective risk communication should present all risk-related information and share it promptly with participants in all aspects of risk communication to correct the knowledge and bridge the experience gaps between experts and the general public's risk perceptions (39).

It is important to understand the media's risk communication mechanism and how the general public perceives risk. Zhang et al. (40) adopted a “risk message-centered” approach to observe and discuss the relationship between the government, media, and the public during the COVID-19, and found that the government and media disseminated risk messages with ambiguous rhetoric and reporting at the early stage of the epidemic, which influenced the public's correct perception of risk facts and, to a certain extent, contributed to the spread of rumors thus increasing panic.

Malecki et al. (41) use perceptions of the general public as a starting point to explore effective risk communication strategies and principles in the modern social media era from the perspective of “danger plus anger”. Christensen and Lægreid (42) analyzed the Norwegian government's communication practices and reputation management performance in the context of the fight against the epidemic from a “crisis communication” perspective. Studies that analyze risk transmission processes and effects through a social trust approach generally confirm that, when individuals trust the government or risk management units, they have lower risk perceptions and are calmer about risk, therefore tending to perceive the risk as manageable (43). In this case, the public is more likely to comply with relevant prevention measures (44).

Conversely, when individuals have less trust in government, scientific reports, and medical professionals, they perceive the risk as highly threatening and are prone to more negative emotions, and may refuse to comply with risk prevention and control measures (45). The “individual perception-action” path (e.g., mental models), which focuses on the factors that influence individual risk perceptions and their behaviors, as well as their influence on the process and effectiveness of risk communication, aims to emphasize the need to fully understand the psychological, emotional, and behavioral dimensions of the public in the risk communication process. The “cultural identity” path (e.g., social network infection), is intended to show that people's risk perception, assessment, communication, and behavioral responses are potentially and profoundly influenced by sociocultural factors such as ideology, values, and ethical norms of the society they live in Huang (46) and Zhang and Ran (47).

With reference to the above-mentioned literature and research paths, this study analyzes the early risk communication mechanisms of COVID-19 by official media accounts and self-media (In China, self-media refers to independently operated social media accounts - on platforms such as WeChat, Weibo, and other smaller ones - usually run by individual users.) accounts in Chinese online communities, investigate the factors that influence people's risk perceptions and preventive behaviors toward COVID-19, and compare the risk communication mechanisms with those of other countries in order to draw more comprehensive and generally applicable effective risk communication experiences, and thus improve the ability of the Chinese government and media to respond to unexpected risk events and subsequent risk communication and management.

## Research Design

This study aims to analyze the communication performance and role of the Chinese official media in the risk event from the perspective of the characteristics of the risk event and the public's perception pathways, to understand what factors influenced the Chinese public's attitude and behavior during the risk event, and what communication role they played in the process. Based on these issues, this study takes the case study of the COVID-19 in China by first conducting an online text analysis, using the social media platforms Sina Weibo and WeChat as the survey method.

Semi-structured interviews were conducted with Chinese social media users to explore the factors that influenced public perceptions and behaviors during the outbreak. In this way, we explored the role of government, media, and listeners in the risk communication process of COVID-19, and provided references for future outbreaks or potential risk communication.

### Method

Sina Weibo is currently the most popular social media and information-sharing platform in China (48). Sina Weibo reaches 523 million active users, with more than 25 million posts per day (Sina Weibo Data Report, 2021), and many official media outlets have set up Weibo accounts to connect effectively with the public. Its usage pattern is similar to that of Twitter in the United States with information mostly disseminated in one direction, and the relationships between users do not start from interpersonal relationships.

WeChat, which has grown and developed based on social relationships, has also increasingly penetrated the daily lives of Chinese people (49). According to official reports, WeChat has over 1.1 billion active accounts (50). Based on its roots in familiar and relatively stable and reliable social relationships, the content posted or shared on the WeChat platform is more easily accepted, trusted, and diffused by the public (51, 52). As China's Xinhua Finance's “Zero Data” monitoring system shows, WeChat groups and WeChat friend circles were the first channels for Chinese people to learn about the COVID-19 (53). On the other hand, Sina Weibo and WeChat are both characterized by their “writing culture” texts, and the strength of their research lies in the authenticity and self-reflexivity of the field, and through the observation of the field, social phenomena and facts can be described in greater depth (54, 55).

Based on the above, the researcher used the early and mid-late stages of the COVID-19 (December 30, 2019, to June 30, 2020; a total of 182 days) as the observation time to collect, organize, and analyze the timing, characteristics, and issues of information released about COVID-19 in the Sina Weibo and WeChat fields. The study then used semi-structured interviews to examine people's perceptions of the COVID-19 and the government's media performance in the online communities with the aim of clarifying the aforementioned online observations and complementing the phenomena and issues not captured by the online observations to enhance the credibility of the study's inferences. Prior to the formal interviews, three respondents were invited to take a test to understand the suitability of the interview protocol and to enhance the sensitivity of the researcher on this topic.

### Research Subjects

In this study, Sina Weibo and WeChat were chosen as the fields of investigation. Sina Weibo mainly used the information on the COVID-19 released by the regional health care committees and 18 mainstream media accounts recognized by the CPC Central Committee, including People's Daily, Xinhua News Agency, Quyi, PLA Daily, Guangming Daily, Economic Daily, China Daily, Central People's Broadcasting Station, CCTV, China Radio International, Science, and Technology Daily, China Discipline Inspection and Supervision Daily, Workers' Daily, China Youth Daily, China Women's Daily, Farmers' Daily, Legal Daily, and China News Agency, as the observation sample, in order to obtain the broadcasting and dissemination characteristics of the government and official media on the COVID-19.

WeChat, on the other hand, used three self-media accounts with high visibility, professionalism, and public trust, namely “Ding Xiang Yuan,” “Fruit Shell,” and “Paperclip,” and five self-media accounts with more than 100,000 subscribers, namely “Magic Girl,” “Mr. Dennis,” “Uncle Guo,” “Listen to the Wind,” and “Koi Youth,” as observation samples to compile the characteristics of social media communication about the epidemic, and to ensure the validity of the information obtained through subsequent interviews with WeChat users.

In terms of recruiting respondents, previous studies have suggested that sociodemographic variables such as gender, education, occupation, and socioeconomic status can have varying degrees of influence on individual risk perceptions and behaviors (50, 56–59). In order to obtain more complete and diverse survey data, the researchers did not restrict the socio-demographic variables of Internet users in the recruitment information. Also, based on ethical considerations, the researcher stated in the recruitment information the identity of the individual, the research question, a summary of the content, the purpose of the study, and the length of the interview required, and stated the measures related to privacy protection. After obtaining respondents' voluntary participation in the interview and signing an informed consent form, a total of 30 respondents were interviewed, of whom 12 were male and 18 were female. Their education level consisted of six completed junior high school, 11 completed high school, and 13 completed university and above. Ages ranged from 20 to 50. Their occupations were University undergraduate and master's degree students (10), teachers (7), housewives (4), media and art workers (6), and industrial and commercial workers (7). Monthly disposable income ranged from 2,500 to 7,000 RMB, and cities of residence were Wuhan (10), Beijing (8), Dalian (7), and Harbin (9).

In the processing and analysis of the interview data, in order to obtain more objective and valid information, after the interviews were processed verbatim, the researcher hired two other researchers to conduct the data coding and analysis, and triangulation was used to analyze and test the data to increase the reliability of the results (60).

The specific steps to analyze the data by the thematic analysis were: (1) word-by-word key concept extraction, i.e., conceptualization based on the relevance of the interview content to the research questions; (2) primary coding, i.e., grouping the interview data into words/phrases/sentences that have substantive meaning and discussion value, in order to derive further conceptualized information; and (3) spindle coding, i.e., to observe, summarize, and classify the categorized contents again to obtain the recurring core concepts.

## Research Analysis

### Communication Performance and Risk Communication Role of Media

#### The “Information Lag” of Official Media Information Release and the Risk Perception and Warning of Self-Media

From the communicator's perspective, the media, as an important source of information and knowledge broker in the risk communication and management process (61), can be an important channel for the public to obtain information about the outbreak, and for the public to connect with scientific findings, arguments, and recommendations (62). The media not only transmits the basic definition and appearance of risk events, but also participates in the construction, negotiation, and transfer of risks (63), and filters and edits the content of information through the rhetorical and reproductive frameworks of epidemic information and news value principles (64) so that it can be amplified during the interactive communication and feedback processes of different communities (65). This affects the original appearance of the risk event and influences the risk perception of the audience (65).

Based on the statistical data of “Knowing Microdata” and the analytical results of the COVID-19 risk transmission life cycle by Zhang (66), the early transmission development stages of COVID-19 can be divided into five stages: latent period, outbreak period, spread period, dissipation period and reignition period. Focusing on the communication performance and risk communication roles of the government and official media, first of all, during the initial period of the outbreak, from December 30, 2019, to January 15, 2020, the regional health committees and 18 official media on Sina Weibo accounts and self-publishing members in WeChat did not show any information related to the COVID-19, but more regular and positive reports in favor of political achievements. The reports were more routine, with a positive performance and praise types of information. The reason for this is that, on the one hand, China's government and official media have always adhered to a top-down “technical model” of technical risks (8, 37, 67), in which the government and official media tend to take a conservative approach in order to avoid causing panic among the population before scientific facts and responses are known, such as the causes of the risk, specific symptoms, modes of transmission, the scope of impact, preventive measures, and related consequences.

On the other hand, the local government hides the risk for the protection of its own interests or political status, while the official media fails to investigate and understand in a timely manner, and generally follows a state of silence in the face of public opinion. The loss of public opinion monitoring and information verification functions not only results in the failure to disclose information about the COVID-19 to the general public in a timely manner but also causes a serious lack of early warning links in the management of risk emergencies, which damage and lower the general public's trust in the official media and cause a series of public opinion incidents, laying the groundwork for subsequent effective risk management.

For example, in the afternoon of December 30, 2019, the “Wuhan WeChat Group” (pseudonym) began to circulate the internal notice of the Wuhan Health Commission about the discovery of a “pneumonia of unknown origin.” The internal notice of the Wuhan Health Commission on the discovery of “pneumonia of unknown origin” was a screenshot of the SARS coronavirus. A WeChat group appeared that night, and the message was forwarded by the self-publishing account “Uncle Guo.” Immediately afterward, the first personal microblog about the epidemic appeared on Sina Weibo which warned, “Don't believe in rumors, don't create rumors, don't spread rumors! But because I don't know if it's true or not, I'd like to try my best to prove it, and I hope it won't cause any anxiety to anyone. But the ‘Wuhan WeChat group’ has gone crazy, so I hope the people who know can come out! Is there really such ‘pneumonia’ in Wuhan?”

Immediately afterward, two more personal microblogs posted articles to verify the authenticity of the “SARS” outbreak, and WeChat self-media accounts such as “Magic Girl,” “Listen to the Wind Wanderer,” and “Mr. Dennis” posted information about disease protection. “Listen to the Wind Wanderer” reported on the “recently appeared about the Wuhan infectious disease news, the official has not yet confirmed[…]but to 2003 SARS as a warning, please pay attention to personal protection, reduce cross-city mobility[…]waiting for official confirmation.” It was not until the afternoon of the next day (December 31) that the official Sina Weibo account of the Wuhan Health and Wellness Commission released the first information about the pneumonia epidemic, confirming its existence of the epidemic. This was followed by dozens of official media accounts, including China Youth Daily, Beijing Daily, People's Daily, Xinhua News Agency, and Quyi, forwarding the news content. This was the official start of risk information dissemination and management.

In summary, the government and official media failed to provide accurate and clear explanations and reports on the risk event at the early stage of the outbreak, failed to give full play to their active role in risk management and communication, and were in a state of information deficiency and lag, which led to the growth and rapid spread of online rumors. In contrast to self-published accounts, which follow a technological model and attempt to achieve a top-down transmission of technical risk information (8, 37, 67), their dominant function and role in active management and dissemination were undermined by the more timely, transparent and comprehensive self-published reports. However, the dominant function of proactive management and communication is undermined by more timely, transparent, and comprehensive self-published reporting. From the perspective of individual-organizational relationships and individual cognition-action, as scientists follow the principle of pursuing objective evidence and continuously verifying research results, they publish risk information with a relative lag, which inevitably generates inconsistencies in information or arguments before and after. The public's trust in the government and the official media is subsequently reduced because scientists are pursuing objective evidence and constantly verifying research results. With the public's proximity to online media and the diversified ways of communication and evidence seeking, the relevant informants through their perception of risk factors change their communication role from being passive information receivers set by the government and the media to active risk communicators, alerting possible risk information on social platforms or self-media accounts and quickly spreading and diffusing it in order to draw the attention of the official media from the bottom up, while the official media, in turn, change their role to that of public opinion receivers and passive communicators.

#### The Linkage of Risk Information Dissemination and Management Between Official Media and Self-Media

On January 20, 2020, the official microblogs of CCTV News, Xinhua Viewpoint, Headline News, People's Daily, and China Daily broadcasted real-time epidemic information in accordance with the instructions of national and local government health and wellness committees, with cases of infection appearing one after another in Zhejiang and Guangdong. That night, CCTV's News 1+1 interviewed Chinese medical scientist and academician of the Chinese Academy of Engineering, Zhong Nanshan, who confirmed that the epidemic was “human-to-human,” a statement which contradicted previous statements made by the Wuhan Health Care Commission, which immediately sparked public outrage. On January 21, Zhong Nanshan held the first press conference on the COVID-19 outbreak in Guangdong and clearly pointed out that there is no effective drug against the virus. On January 23, Wuhan was “closed,” which led to the global community's attention to the outbreak. On January 21, rumors started to spread on online media platforms, mixing true with false information. On January 28, the official microblog of the Supreme People's Court took the lead in vindicating the eight “rumor-mongers,” stating that “if the law is applied mechanically, it is indeed possible to conclude that, given that COVID-19 is not SARS, the Wuhan SARS epidemic has been reported. The emergence of SARS in Wuhan is a fabrication of false information, and the information has caused social disorder, which is in line with the act of fabricating and spreading false information as stipulated by law, and it is justified to give administrative punishment or even criminal punishment. However, it has been proven that although COVID-19 is not SARS, what the information publisher posted was not a complete fabrication. Had the public listened to this ‘rumor’ at the time and taken measures such as wearing masks, strict disinfection, and avoiding further visits to wildlife markets based on their fear of SARS, it might have helped citizens of Wuhan to better prevent and control COVID-19 today. However, the inconsistency of such statements led to the public's search for the “truth” and the vindication of public opinion across the Internet reached a peak. On January 30, the Hubei Provincial COVID-19 Prevention and Control Command held a press conference in which Jiang Chaoliang, then secretary of the provincial party committee and head of the provincial COVID-19 and Control Command, answered reporters' questions. When asked by a CCTV reporter about the shortage of medical supplies at the Union Hospital, Jiang Chaoliang provided a scripted answer prepared in advance, further stirring up public anger, the online media also scrambled to discuss the matter. On February 3, the Political Bureau of the CPC Central Committee began to punish officials for dereliction of duty, focusing on problems such as “telling lies and reporting false information,” “eagerly painting slogans, shouting slogans, and making statements,” “reporting good news to superiors but not to the public,” “responding passively and ignoring human lives,” replacing personnel in relevant positions, and vigorously managing the management ecology of the officialdom.

At around 10:00 p.m. on February 6, a statement from Wuhan Central Hospital reported that Dr. Wenliang Li, who had tried to warn the public about COVID-19, died of the disease. News of his death began to circulate on WeChat's “Ding Xiang Yuan,” “Paperclip,” and “Fruit Shell” public accounts, as well as several WeChat groups and personal circles of friends. In the early morning of February 7, People's Daily, CCTV News, China News Network, and the official Weibo account of Yao Chen, a well-known film and television actress on the mainland, posted a message: “Expect a miracle,” but at around 4 a.m. on February 7, the Wuhan Health Care Commission announced on its official website that Dr. Li had died. The incident immediately ignited public sorrow and anger, inspiring people to “accuse” officials of negligence and dereliction of duty. Subsequently, China's National Supervisory Commission sent an investigation team to Wuhan to “conduct a comprehensive investigation into the issues related to Dr. Li Wenliang as reflected by the public. At the same time, videos of the medical environment of Wuhan Hospital and the admission of cases began to appear on WeChat, TikTok, Weibo, and other platforms, deepening people's fears and causing chaos around the country as they snapped up medical supplies and medicines.

Statistics from “Zhiwei Data” show that between February and March 2020, official media and self-media risk information content on Weibo and WeChat aimed to broadcast the incidence of infections, medical treatments, and the construction of related medical supply and temporary treatment facilities in various regions. At the same time, the platforms also began to focus on the management of online rumors and the establishment of fact-checking platforms.

In summary, it can be seen that the government's management and communication strategy, under the influence of media opinion, gradually became took a corrective direction, accountable to relevant managers, unified in information on various platforms, and unified in content. The government's management and communication strategy, under the influence of media opinion, gradually became a mechanism and management role to correct the direction, hold accountable the relevant managers, unify the information on various platforms, stabilize public sentiment, and strengthen and improve effective risk management. Even though there are still cases of infection and the epidemic has not yet been truly quelled due to the continuous mutations of the virus and global population movements, media platforms such as TV, official online media accounts, microblogs, WeChat, and other government risk management units can learn from the previous experience of risk communication by broadcasting timely, open, and transparent reports on the risk situation, prevention measures, and management effectiveness, and form a joint online and offline broadcast to correct the content of wrong news.

In general, the government and official media accounts showed a “lag-broadcast-correction-accountability-unification” communication mechanism in the early dissemination and management of outbreaks. The technical model of communication management has led to inconsistencies in the official media's presentation of information about the epidemic, which has reduced public trust and led to a rise in collective negative emotions. However, due to the characteristics of social media, which are highly usable, low barriers to professional entry, immediate, decentralized, dynamic, and fast dissemination, and the ability to break through the qualitative model of traditional media production, dissemination, and control, social media became the main channel for public access and dissemination of epidemic information.

Throughout the COVID-19 pandemic, personal and niche media microblogs and WeChat accounts (especially WeChat) dominated in various aspects of risk warning, risk dissemination, and public opinion guidance (68). In other words, the official media's original active communication and message control shifted to social media, and the official voice and an active communication role also shifted to social platforms and the public, resulting in the proliferation of fake news and unstable public sentiment at the beginning of the epidemic, as well as chaotic incidents such as drug and mask grabs and police beatings. The specific communication mechanism of this risk event in the self-media can be summarized into three stages. First of all, the social media “awareness, early warning, and diffusion” outbreak information, “from the bottom up” to attract the attention of mainstream media, reports, and official notification; Secondly, using the scientist (Zhong Nanshan)'s speech and risk contrast (SARS) and the strategy of “fear appeal,” the intention is to stimulate people's risk perception and emotional reaction, and then enhance interpersonal communication, discussion, and network forwarding effect. Finally, with the epidemic slowing down and the government's flexible use of risk management and risk information delivery forms and channels, the self-media and the official media formed a dynamic cycle of “interaction-union-error correction,” and completed a complete closed-loop of risk information dissemination. As Hua and Shaw (2020) found from an analysis of data on outbreak-related information in Chinese newspapers, social media, and other online platforms, despite China's late response to the outbreak, risk management units and media were able to identify communication and management gaps and by effectively combining the advantages of big data and online platforms strengthen online information censorship and regulation, and promote the responsibility and effectiveness of individual action and the effectiveness of collective protest through the Internet, thereby calling on the public to comply with individual and collective rules for epidemic prevention and contributing to effective risk management.

### Public Communication Roles and Their Risk Perceptions and Behaviors

#### Risk Similarity and Spatial Proximity Affect People's Risk Perception and Behavior (Communication, Risk Protection)

The risk perceptions and risk judgments of the general public are easily influenced by the memorability of past events and the imaginability of future events (69). During the spread of the new epidemic, people were more likely to associate themselves with the SARS risk event in 2003, and because of the initial uncertainties and information gaps in this risk event, people were more likely to overreact and rush to buy medical supplies, forward related information to friends and relatives without checking or ignore risks, and other wrong risk perceptions and inappropriate behaviors. For example, Mr. Zhang (male, 37 years old, interview time: 2020.02.14), an architect living in Wuhan, said:

“Discussions and photos of COVID-19 in the hospital appeared in the WeChat group. I didn't believe it at first, so I went to Weibo to check...ask friends who work in the hospital...and then forward relevant information to friends, just like during the SARS before. If you really wait for the official notification, nothing will be left...”

Ms. Ye (female, 42 years old, interview time: 2020.02.17), a housewife living in Beijing, said:

“When I heard that there were suspected cases in Wuhan, I immediately thought of SARS in 2003. I was a little scared, so I bought masks regardless of whether it was true or not...”

Ms. Li (female, 48 years old, interview time: 2020.01.22), a University teacher living in Dalian, said:

“I first saw SARS in Wuhan in the WeChat group. Although I felt that Wuhan was far away from here, I might not be affected, but I still bought three packs of masks and then told my friends to buy some, too. Everyone would rather believe it and don't be like before (SARS). When it really comes, I can't find masks again.”

There are also people who initially considered that the distance between their place of residence and the place where the risk occurred was far, and so ignored the existence of the risk. They did not take any preventive measures and believed that they were less susceptible to infection. The “third-party effect” provides a good explanation for the emergence of this phenomenon, that is, people believe that risk has more influence on others than on themselves. In other words, others are more likely to be infected through risk-taking. For example, Ms. Li (female, 33 years old, interview time: 2020.02.20), a freelancer living in Harbin, said:

“There are a lot of fake news on the Internet now, even if Covid-19 is true, it may not reach Harbin. Like SARS, it is too cold here, the virus came and froze to death, so i really didn't take it seriously at the beginning”

#### Fear Appeals Tend to Weaken the Efficacy of Risk of People

While the public needs to be informed about risks, the presentation of risk messages can lead to fear and pessimism. The mere mention of the adverse effects of risk issues (no matter how small the probability of their occurrence) by communication agencies or personnel in the risk communication process can increase people's perception of the probability of risk and increase their fear of risk (71). In other words, while the use of fear appeals to disseminate risk information may enhance risk prevention and protection behaviors to a certain extent, it also tends to increase negative emotions and psychological stress due to misinformation overload and loss of efficacy (72). For example, Ms. Li (female, 29 years old, interview time: 2020.03.01), who lives in Beijing and works in the business service industry, said:

“I often read relevant information on TikTok, WeChat, and Weibo. The information about COVID-19 on TikTok scared me, and they used that kind of scary soundtrack, you know...On the map, the cities and the number of cases gradually turned into dark red, which was terrifying...Sometimes I was really desperate. I felt that the earth was about to be destroyed. No matter how much we did, it would not help.”

Mr. Jia (male, 24 years old, interview time: 2020.02.19), a college student living in Wuhan, said:

“I usually learn about the COVID-19 information through online forums, games, Weibo, WeChat, and TikTok. I have been depressed for a long time...”

Ms. Wang (female, 21 years old, interview time: 2020.03.07), a college student in Harbin, also said:

“Every day I turned on my mobile phone and computer, and there were more infections and deaths. Horrifying pictures and music are everywhere. The school start date was always uncertain, and it was very annoying...”

And Ms. Guan (female, 46 years old, interview time: 2020.02.19), a university teacher living in Beijing, said:

“I usually learn about the COVID-19 information on Toutiao, Sina News, or on TV. I don't really believe the content on social media, even though the information was updated quickly. It will inevitably be one-sided or even wrong. On TV and some news apps, the news was relatively objective, and it can be seen that the country put efforts into the pandemic and what specific work and results have been done. On the one hand, I can understand the immediate information, and on the other hand, I can understand what needs to be done to prevent it. Therefore, during this period of time, I could not say I was optimistic, but still relatively stable.“

And Ms. Ye (female, 37 years old, interview time: 2020.03.04), a high school teacher living in Dalian, was also mentioned.

“……There are a lot of fake news on the Internet, and it is not easy to distinguish. It is better to watch TV directly to know about the relevant content.”

This also suggests that risk communication is easily influenced by the form of presentation or discursive framework (69). When people lack a strong original viewpoint, they are easily dominated by the presentation of messages. For example, social media platforms more often presented fear appeals, and simplistic presentation of risk effects in the early stages of an epidemic with shocking music effects, which could easily cause an increase in acute stress disorders. It is also noteworthy that, because of the social and cultural factors, the risks of the situation were not always easily understood. The low threshold of content production and the lack of a rigorous and comprehensive censorship mechanism in self-published media can easily become a breeding ground for fake news and hinder effective risk communication. In contrast, official media accounts and traditional media, especially television, focus on the description of the effectiveness of risk management in the process of disseminating risk information, which helps to enhance people's trust in the state and government's ability to manage risks and helps to stabilize public sentiment without causing mass panic.

#### Relevance and Social Norms Influence the Risk-Protective Behavior of Regular Citizens

People are more likely to be aware of direct or personally relevant risk threats. Personal experience, observation or knowledge, spatial proximity, and duration of residence are all related to risk judgment and assessment (64). In this outbreak performance, people were more likely to raise their risk awareness and make behavioral changes, such as wearing masks, washing hands regularly, and disinfecting touched objects with alcohol, in the event of a local case of infection. For example, Ms. Wang (female, 39 years old, interview time: 2020.03.14), a media worker living in Harbin, said:

“Although I felt that the pandemic might be serious at first, I thought there was also a chance that COVID-19 would not spread to a place as far as Harbin. Since everyone didn't wear masks at first, I didn't do anything deliberately or take it seriously. Later, there were cases of infection in Liaoning, Jilin, and Harbin, and the range of activities of the infected people was wide, so I just started to wear masks.”

Mr. Li (male, 36 years old, interview time: 2020.03.09), an art worker living in Beijing, said:

“At the beginning, I didn't wear a mask all the time, because I often forget it, and I think it's okay not to wear it occasionally. Later, there were more and more people reminding me to wear a mask, and indeed there was an outbreak in Beijing, so I began to pay attention to personal protection.”

Another point that can be drawn from Mr. Li's answer is the influence of community and social norms. Further, an individual's behavior is easily influenced by the attitudes, language, and behavioral norms of those around him or her. As the number of people who engage in a certain norm of behavior increases, individuals will change their behavior to meet this personal and social need out of a desire to fit in and not be excluded.

For example, Mr. Ji (male, 22 years old, interview time: 2020.03.11), a college student living in Harbin, mentioned that:

“...You are required to wear a mask everywhere. If you don't wear it, others will stare at you. Even if you don't say it, you will definitely be criticized in their mind...”

Mr. Wang (male, 21 years old, interview time: 2020.03.11) a student from Dalian also said:

“...I sometimes forget to wear a mask when I go out. After all, I am not used to it, but when I go out and see everyone wearing a mask, I will go home to get it... ”

Some interviewees also said (Ms. Ma, Dalian, female, 22 years old, interview time: 2020.03.06):

“In places with less people, I will take off the mask to breathe, or take pictures. How to take pictures with the mask on? Sometimes I think taking it off is okay, but sometimes I feel embarrassed. However, as long as I'm not embarrassed, it's someone else who is embarrassed…”

To sum up, risk imaginability and recall, personal relevance, spatial proximity (distance), media framing, communication strategies, and social norms can all significantly influence people's risk perceptions and behaviors in the early stages of a risk outbreak. If the government and the media fail to inform, publicize, and educate in time at this stage, it may cause fear and a negative response. However, it should also be noted that the style of media reporting and the recurrence of risks may have a counterproductive effect on the public's psychology and emotions. As stated in the Extended Parallel Process Model, on the one hand, fear appeals can help stimulate the public's positive response awareness and behavior, while on the other hand, it may also cause people to lose their sense of perceived risks, being unable to cope with the obstacles or ignoring the impact of risk (70).

From the perspective of the guidance of social norms on behavior, social norms have a positive effect on individual behavior, but gender and personal values may also affect social norms on it to a certain extent. It is generally confirmed in the literature regarding numerous health and environmental risks that gender, age, income, education level, and values will significantly affect personal risk perception and behavior (50, 56–58). Focusing on the analysis samples of this study also showed the same results. Women around the age of 40 showed a higher risk perception in this pandemic, that is, they believed that Covid-19 was a high risk. Respondents with a higher education level (above university) were more likely to search for risk information, and understand and disseminate risk-related information more rationally.

## Discussion

The purpose of this study was to analyze the characteristics and effectiveness of official Chinese media communication about COVID-19 on two popular social media platforms, Sina Weibo and WeChat, between December 30, 2019, and June 30, 2020, to determine how this communication affected people's risk perceptions and protective behaviors. The results from the analysis of this study show that the dissemination of COVID-19 information fully demonstrated the characteristics of public health emergency communication, and how it is different from other forms of crisis communication or emergencies in terms of content and intensity. This risk communication combined important factors of medical research such as emergencies, natural disasters, emergency relief, highly infectious threats, and uncertainty, presenting a different unknown risk and unstable communication environment and mechanism (66).

### While the Government and Official Media Were in a State of “Lack of Information and Lag” at the Beginning of the Outbreak, Self-Media Played a Key Role as a Risk Perceiver

The government and official media have always adhered to a technical model aimed at managing and disseminating risk information from top to bottom. This study found that at the beginning of the COVID-19 epidemic, the official media accounts experienced “information lag” deficiencies because the risks had not been confirmed by the scientific community and effective defensive measures had not been identified. The delayed reporting and negligent management of the epidemic by local risk management units led to the failure of the government and official media to perform their risk warning. The official media failed to perform the power and function of risk warning and timely control. In addition, the official media did not open effective channels of dialogue between the government and the public, and the control of public opinion and rumors on the Internet lagged behind.

In contrast, self-media, by virtue of its proximity, low professional access threshold, instant, decentralized, dynamic, and fast dissemination characteristics, successfully broke through the qualitative mode of traditional media production, dissemination, and control, making themselves the main channel for the public to obtain and disseminate epidemic information on COVID-19. The individual and niche Weibo and WeChat accounts (especially WeChat) became the main channel for risk warning, risk dissemination, and public opinion guidance, playing the key role of “risk perceiver.”

However, in this process, it was difficult to guarantee and control the accuracy and authenticity of circulating information, because unverified information spread at an uncontrollable rate (72), and the public was influenced by a large amount of mixed information, making it difficult for them to distinguish between scientific evidence and unreliable information (73). As concluded by Dubey et al. (74) and Pedrosa et al. (72), information overload and indistinguishable outbreak content exacerbate public anxiety. This study also found that self-published media often amplified information related to the epidemic through fearful music, images, and text during the epidemic, further exacerbating the public's panic and anxiety, resulting in a loss of efficacy and consequently a negative response to the risk.

In this regard, this study suggests that future risk communication and management needs to pay more attention to social opinion and public risk perceptions and emotions, disclose all information about outbreak risks in an adequate and timely manner, and ensure social warning, social mobilization, and effective cooperation between social agents and the government. At the same time, in addition to using fear appeals to raise public alertness to risks, official media should provide necessary and effective risk response measures to ensure a high sense of public efficacy, and appropriate control of self-media content to avoid the spread of false news in cyberspace and, consequently, into the greater public sphere.

### During the Middle and Later Periods of COVID-19, the Government and the Official Media Began to Pay Attention to the Influence of Self-Media on Peoples' Emotions and Behavior, and Gradually Improved the Supervision of Online Information and the Operation of Official Media Accounts. This Was Intended to Achieve an Information Consistency and Linkage Mechanism Between Official Media and Self-Media, to Prevent and Correct Mistakes

In the middle and late stages of the epidemic, the official media gradually paid attention to timely and effective communication with the public and fully operated official media accounts, shaping a multi-channel collaborative release and risk management posture of official and self-published media. This study found that the official media were influenced by the content of self-published media and public opinion, and gradually set up a mechanism to check false information and control the content of self-published media, which led to a timely and accurate announcement of the epidemic by official media. At the same time, public panic and anxiety were alleviated, resulting in good social effects.

In addition, under the control of the official media, the information on official mainstream and social hotspots increased. Although there were still flaws in the excessive use of fear appeals to gain more traffic, in general, the content of the self-media gradually tended to be rational, and the public began to notice the unchecked characteristics of the self-media information, and thus gradually regained trust in the official media.

On the whole, COVID-19 revealed the weaknesses and deficiencies of the Chinese government in public health, government governance, and social systems. The government was not sufficiently alert to self-media rumors and sudden risk information and failed to grasp and manage the network opinion in time and establish an effective information checking mechanism. However, these shortcomings can also be effective entry points to promote social construction and reform. As Sun (75) suggests, SARS and COVID-19 were two major events that forced reforms in China's public health system, with SARS correcting the direction of public health reform and promoting the reconstruction of the public health system, while COVID-19 improved the Chinese government's disease prevention and control system and mechanism, as well as the social governance system for major public health emergencies.

From the perspective of information communication governance, public health emergencies are no longer just a health system issue, but a global issue concerning the modernization of national information governance and governance capacity. Information governance is different from the administrative governance of information. In the traditional emergency governance practice, the government is always regarded as the only source of emergency prevention and control and bears the entire responsibility of emergency governance (76). In the era of new media and mobile communication, administrative power alone can no longer cope with risk crises, and risk crisis management relies more on the participation, cooperation, and collaborative governance of multiple actors such as government, media, social organizations, enterprises, and citizens (77).

The communication governance of risk crises should ensure effective dialogue among multiple actors, and in effective communication, promote truth restoration and interest remediation, as well as rebuild trust (78). Furthermore, building multi-level crisis management and risk management paths such as information releases, crisis management, and public opinion guidance to realize multi-level dialogue, are needed to turn risk crises into opportunities for effective risk management and social governance (77).

### When Faced With an Unexpected Health Risk, the Public's Recall of Past Risk Events, the Relevance of the Risk to the Individual, Spatial Proximity (Distance), Media Framing and Communication Strategies, and Social Norms Can All Significantly Influence Their Perception of the Risk and Related Behaviors

In-depth interviews with audiences revealed that social norms were the main factors influencing public self-health management behavior in this risk event.

First, the risk event reminded the public of SARS, which led to panic and a rush to buy medical supplies before the risk event was officially confirmed. Then, the fear appeal communication strategy favored by the self-media further aggravated the public's fear and pessimism, leading to a decrease in their self-efficacy. In order to stabilize public sentiment, official media tried to avoid using fear appeals when managing risks and broadcasted the current situation of risks and protective measures more objectively to enhance the public's sense of efficacy in self-health management. The public's perceptions of the severity of the epidemic and self-inflicted diseases differed from region to region. Therefore, social norms held an important influence on public self-health management in the middle and late stages of an epidemic, with the behavior and evaluation of others significantly influencing public behavior. In this regard, this paper suggests that the differences in public perceptions of health risks should be taken into account and that social norms should be used to guide public behavior in risk communication and management so that risk communication and opinion guidance can be carried out in a more targeted and comprehensive manner.

### Limitations

Although this study conducted in-depth interviews on the perceptions and behavioral influences of some online Chinese citizens, it did not interview government-related units or other official media to ascertain the reasons for the hindrances we found in effective governmental risk management. Also, this study only analyzed the online texts of 18 official media accounts and did not conduct more in-depth data mining. Future research may benefit from using big data to explore and more comprehensively analyze and organize the differences and similarities between official and private information dissemination in sudden risk events.

## Conclusion

### The Government Should Strengthen the Use of Social Media, Rumor Controls, and Fact-Checking Mechanisms, and Maintain Timely Communication With the Public

The use of self-media (WeChat, Weibo, et al.) may be an effective channel for the government and related agencies to communicate immediate and accurate information to the public in times of risk and crisis (79). On the one hand, relevant government departments should strengthen the operation and maintenance of online social platforms, provide transparent and timely updated risk information, and analyze the collation and analysis of public opinion, so as to actively anticipate and respond to the possible social impacts and fluctuations of identified risks.

It is important to ensure timely, and comprehensive communication and experts and decision-makers should fill in the information gaps among other organizations and individuals. This way, in addition to avoiding the confusion and influence of rumors or fake news, the public will be informed and equipped with the correct knowledge on how to protect themselves and those around them in the event of an unexpected risk event.

Misinformation or rumors can easily spread widely on social media and may increase people's perception of risk and fear of health-related topics (16, 80, 81). This makes it imperative for relevant governmental functions and community platforms to publish and regulate online information. The relevant government departments should also establish a sound online fact-checking platform and inform the public through diversified multi-channel to strengthen the public's attention and use of this platform so that the public can obtain it accurately. At the same time, strengthening the dialogue among functional departments, regional governments, academia, and civil society will ensure the implementation of effective risk management policies, help to clarify public concerns, and prevent obstacles to policy implementation.

### The Media Should Pay Attention to the Timely and Accurate Reporting of Risk Events, as Well as the Presentation of Information on the Causes, Modes of Transmission, and Risk Impacts, and Be Cautious About Possible Social and Psychological Impacts of Risk Reporting, so as to Enhance Public Trust in the Government and the Media

In public health and health risk situations, the public is often in need of up-to-date and accurate risk information and advice on risk management to better protect themselves and their friends and family. The media serves as an important bridge for public risk perception (82), and the public's reliance on the media may be even stronger during unexpected risk events (83). The public expects the media to report risk assessments and give sound risk response advice through authoritative and trustworthy sources (79). Effective and accurate reporting of risk facts by the media can significantly increase the accuracy of public perception of risk (84), but with fragmented or scarce risk information and inconsistent and uncertain multiple discourses (government, experts, media, etc.), rumors spread through the Internet to various fields, resulting in increased negative emotions such as fear, worry, and anxiety among the public (85, 86). People are more likely to overestimate certain threat factors that are less risky or underestimate certain factors that are riskier, thus creating one-sided or false risk perceptions (87). People may even engage in disordered or overly aggressive risk responses and violent emotional outbursts, such as hearing rumors that spraying alcohol on a mask can increase the mask's viral defenses, or verbally abusing or beating a person suspected of being infected with the virus. Especially when the inherent uncertainty of risk factors is combined with the ambiguity of message communication, the public's psychological stress increases and negative emotions rise, thus accelerating the spread and proliferation of rumors. Therefore, the public should be better educated and advised to trust and rely on authorities such as the National Health Council, Centers for Disease Control and Prevention, or World Health Organization for the latest information on disease prevention and transmission and community-level threats.

It is also critical for the media to disseminate information to the public in order to promote appropriate health-protective behaviors and effective institutional responses. The media should not use sensational or distracting images when disseminating information to avoid paranoid behavior by the public. In addition, communicating risk prevention knowledge and actions that can be taken to promote changes in behavior should be done in layman's terms and in a manner that is clearly understood and accessible to all.

### The Public Should Enhance Risk Information Recognition and Dissemination Literacy to Avoid Further Spread of Rumors

Although global pandemics have occurred many times throughout history, the emergence and popularity of social media, has taken on an important role in educating the public on how to properly access, analyze, create, and effectively communicate risk information or other messages. However, through the process of dissemination and management of the epidemic, it is clear that misinformation about COVID-19 on online social media platforms adds to the confusion. Because the media access rights of online citizens were expanded with a low threshold, and convenient easily disseminated video and image production were available on social media platforms, fear and anxiety spread through cyberspace. This not only greatly reduces the public's trust in the government, but also hinders the effective management of risks. Therefore, media literacy should be considered a priority for prevention, mitigation of virus transmission as well as risk management, and necessary preparation for health management units to respond to risks in situations requiring rapid response (88).

All countries, governments, and relevant authorities should strengthen investment in and development of citizens' media literacy to help people learn early about disease management, infection prevention, effective dissemination of risk information, and social responsibility, as well as the potential social impact of their online behavior. At the same time, parents, schools, organizations, and even communities should actively conduct media literacy promotion activities to assess individual media literacy situations and issues, keep abreast of citizens' Internet use and problems and eventually establish rational information dissemination habits and atmosphere.

## Data Availability Statement

The original contributions presented in the study are included in the article/supplementary material, further inquiries can be directed to the corresponding author/s.

## Author Contributions

LP: conceptualization, methodology, and validation. ZF: data curation, writing original draft preparation. ZQ: writing review and editing. All authors contributed to the article and approved the submitted version.

## Conflict of Interest

The authors declare that the research was conducted in the absence of any commercial or financial relationships that could be construed as a potential conflict of interest.

## Publisher's Note

All claims expressed in this article are solely those of the authors and do not necessarily represent those of their affiliated organizations, or those of the publisher, the editors and the reviewers. Any product that may be evaluated in this article, or claim that may be made by its manufacturer, is not guaranteed or endorsed by the publisher.
